# Enhancing Academic Success: A mixed Study on the Influencing Factors among Pharmacy Students in Syrian Universities

**DOI:** 10.12688/f1000research.151218.2

**Published:** 2024-10-11

**Authors:** Mohab Qattan, Mayssoon Dashash, Zeina S. Malek

**Affiliations:** 1 Medical Education, Syrian Virtual University, Damascus, Damascus Governorate, Syria; 2 Faculty of Dentistry, Damascus University, Damascus, Damascus Governorate, Syria; 3 Faculty of Dentistry, Syrian Private University, Damascus, Damascus Governorate, Syria

**Keywords:** Academic Achievements, Academic performance, Pharmacy students, Mixed-methods study, Quantitative phase, Qualitative phase.

## Abstract

**Background:**

Academic achievement is the result of both effort and perseverance exerted by the students. This mixed-methods study aims to investigate the factors affecting the academic achievement of pharmacy students in Syrian universities.

**Methods:**

A convergent parallel mixed-methods study was utilized. In the quantitative phase, a cross-sectional study was conducted on 1008 students (773 females and 235 males) from 23 Syrian universities. A questionnaire consisting of 48 items was designed to be completed by pharmacy students using a 5-point Likert scale.

In the qualitative phase, twelve questions were developed to interview thirty pharmacy students from five Syrian universities to obtain in-depth insights into the factors influencing their academic achievement.

**Results:**

A significant number of students lacked effective time management skills, identified as a weakness among students. The majority of students faced challenges in maintaining a consistent study routine, averaging a score of (2.0).

Motivation towards learning emerged as a crucial factor in enhancing academic performance. Lecturers in the pharmacy faculty employed traditional teaching methods (2.01), and the pharmacy curriculum was perceived as lacking modernity (1.92).

Quantitative findings demonstrated that pharmacy students experienced exam-related anxiety (2.05), identified as a weakness in the qualitative phase.

Factors associated with the Syrian crisis, like unreliable electricity (1.87) and transportation issues (1.83), could have an impact on academic achievement. Economic conditions were identified as challenging to students’ academic performance, negatively affecting the learning process (1.98).

**Conclusion:**

The results of the study demonstrate that personal factors, lecturers, educational environment, exams, and the Syrian crisis influence the academic achievement of pharmacy students in Syrian universities.

## Introduction

Academic achievement serves as a measure of students’ attainment of their academic and educational objectives.

The enhancement of students’ academic achievements has consistently been a primary goal in the field of education. ^1^

In the context of this study, academic achievement is defined as students’ ability to meet the academic standards and competencies required for success in their respective field. Specifically, for pharmacy students, it encompasses not only their performance in examinations and coursework but also their preparedness to deliver pharmaceutical care effectively. This includes mastering both theoretical knowledge and practical skills critical to patient care and public health.

Within the pharmacy sector, academic achievement holds great significance as it reflects a student’s competency and readiness to practice pharmacy effectively and safely.

Graduates are expected to deliver appropriate pharmaceutical care to patients and communities, ^2^ shaping the competency and caliber of future pharmacists. ^3^

Previous studies have indicated that the academic achievement of pharmacy students is influenced by various factors. ^4^^,^^5^ These factors are student-related such as effective time management, ^5^^,^^6^ strong family support, ^4^^.^^6^^,^^7^ motivation toward learning, ^4^^,^^8^ as well as lecturer-related factors, ^7^ such as their skills, dedication, relationship with students, and involvement in the learning process. ^4^

Dealing with academic challenges such as low grades, difficult assignments, and exam pressure is a key factor for achieving success in pharmacy education. Studies have shown that the ability to cope with these difficulties and overcome them is positively associated with academic achievement and success. ^4^ Research highlights the importance of developing effective coping mechanisms and having social support to ensure successful academic outcomes, particularly in the face of stress associated with intensive educational programs. ^4^

Further studies have indicated that strategic studying ^9^ and learning styles ^10^ could significantly enhance academic achievement.

Moreover, the educational environment and university-related issues also play a significant role. ^11^ The quality of classrooms, laboratories, technological resources, and the curriculum itself ^12^ all contribute to student performance improvement.

Overall, ensuring an optimal academic environment by addressing these factors could lead to significant enhancement in academic achievement in pharmacy education.

The impact of exams influences academic achievement. ^9^ Students’ anxiety levels towards exams, the perceived quality of exams within the pharmacy faculty, the effectiveness of exams in assessing knowledge, and their motivation to achieve high grades are key contributors to their success. ^5^^,^^9^^,^^13^

The Syrian crisis has greatly affected pharmacy students, presenting them with additional challenges amidst challenging circumstances. ^14^

The current situation in Syria requires highly professional and competent personnel to respond to the population’s needs and deliver optimal care. ^15^

One is that remains unexplored is the perceptions of pharmacy students in Syria regarding the factors shaping their academic progress. The increasing concern over low academic achievement among pharmacy students in Syria underscores the urgency for a comprehensive investigation into the underlying factors that contribute to this issue.

This mixed-methods study aimed to investigate the factors that affect the academic achievement of pharmacy students in Syrian universities.

## Methods

### Study design

A convergent parallel mixed-methods study was conducted between March and June 2023 to explore the experiences of participants and investigate the factors affecting the academic achievement of pharmacy students in Syrian universities. Both quantitative and qualitative data were collected and analyzed separately within a concurrent timeframe, followed by a phase of integrative evaluation of the collected findings.

### Ethical considerations

Ethical approval for this study was obtained from the Ethical Committee of the Syrian Virtual University (Approval Number: 233/o, Date: 13/2/2023). This study adheres to the ethical principles outlined in the Declaration of Helsinki.

Informed, written consent was obtained from all participants prior to completing the survey forms in the quantitative phase, and verbal consent was recorded before conducting the interviews in the qualitative phase. Participants’ information and data were treated with strict confidentiality, ensuring that personal details were anonymized. The collected data were securely stored on a password-protected computer, and measures were taken to dispose of the data appropriately once it was no longer needed.

The decision to use verbal consent instead of written consent in the interviews was approved by the ethics committee based on the following reasons:

Enhanced participant comfort and flexibility: Verbal consent was considered more comfortable for participants and provided greater flexibility in the study participation process, as they were not required to sign a written document.

Preservation of participant anonymity: Obtaining verbal consent was the preferred option to maintain participant anonymity and minimize potential risks associated with written documentation.

The use of verbal consent was ethically approved by the committee, and all necessary precautions were taken to ensure the confidentiality and protection of participants’ rights.

The quantitative phase of the study was conducted and reported in accordance with the Strengthening the Reporting of Observational Studies in Epidemiology (STROBE) guidelines. ^16^

The qualitative phase was designed and reported according to the Consolidated criteria for Reporting Qualitative research (COREQ). ^17^

### Participants

In this study, specific inclusion and exclusion criteria were established to ensure that the selection of participants aligned with the research objectives. The sample was collected from pharmacy students from the second to fifth years, with first-year students excluded from participation as the first year is preparatory for all medical sections. The information collected from participants included gender, university name, and academic year.

Participants were selected based on their academic year (second to fifth year), program specialization (pharmacy), and active enrollment in a recognized pharmacy program in Syrian universities.

Both public and private universities were included to provide a comprehensive representation of the academic landscape. Geographical limitations were minimized by involving universities from different regions in Syria, ensuring a diverse range of student experiences and perspectives.

These criteria were designed to enhance the study’s validity by ensuring consistency in data collection, and to accurately represent the target population of pharmacy students across Syria.

In the quantitative phase, a cross-sectional study was conducted to investigate the factors influencing the academic achievement of pharmacy students across Syria. Data collection involved the distribution of an online survey to Syrian pharmacy students through websites and social media platforms. The targeted sample size was approximately 1300 students from different Syrian universities. A total of 1008 pharmacy students from 23 Syrian universities participated in this study, resulting in a response rate of 77.5%. In the qualitative phase, data were collected through face-to-face, semi-structured interviews carried out with thirty pharmacy students from five Syrian universities to explore student perceptions of factors affecting their academic achievement. All selected participants in this phase completed the interviews. The research objectives were explained to them, along with their role in achieving those objectives.

### Instrument of measurement

In the quantitative phase of the study, a questionnaire consisting of 48 items was designed to investigate and identify the significant factors that affect the academic achievement of pharmacy students in Syrian universities.

To ensure clarity and validity, all statements within the questionnaire were kept simple and clear. Additionally, three academic members reviewed the questionnaire to assess its clarity and relevance of the statements in alignment with the study’s aims.

The authors selected the academic members based on their expertise and experience in the field of pharmacy education and research. Criteria for their selection included:

Subject Matter Expertise: The experts were chosen for their in-depth knowledge and experience in pharmacy education, curriculum development, and assessment methods to ensure they could provide valuable insights into the clarity and relevance of the questionnaire statements.

Research Experience: The selected experts had a background in educational research, particularly in the area of academic assessment and evaluation, to ensure they could critically evaluate the questionnaire in alignment with the study’s aims.

Understanding of Study Aims: The experts were chosen based on their understanding of the specific aims and objectives of the study to ensure that their feedback on the questionnaire’s content and face validity would be relevant and aligned with the research goals.

By selecting experts who met these criteria, the authors aimed to ensure that the questionnaire underwent a thorough evaluation for both content and face validity by individuals with the necessary expertise and knowledge in the field of pharmacy education and research.

In the initial design, the questionnaire contained 52 items. However, based on the feedback by the academic experts, 6 items were removed due to redundancy or ambiguity, while 2 new items were added to better cover the educational environment and exam factors. Additionally, several other items were modified to improve clarity and ensure the questionnaire was fully aligned with the research objectives.

The questionnaire items were categorized into five sections, each focusing on distinct factors; 8 items related to student factors, 10 items related to the lecturer factors, 15 items related to educational environment, 8 items related to exam factors, and 7 items related to factors associated with Syrian crisis.

The internal consistency of these factors was evaluated using the alpha Cronbach test.

Within the questionnaire, 27 items were phrased negatively requiring reverse scoring to maintain consistency in the directional interpretation across both positive and negative items.

A Likert five-point scale was used with the scale representing: 5: Strongly Agree, 4: Agree, 3: Neutral, 2: Disagree, 1: Strongly Disagree. The results, presented in Table 1, were interpreted to assess the influence of each factor on academic achievements weaknesses.

**Table 1.** Weighted Mean for 5-Point Likert Scales.

Weighted mean	Result	Result Interpretation
1 to 1.8	Strongly Disagree	Very Influential
1.81 to 2.60	Disagree	Influential
2.61 to 3.40	Neutral	Neutral
3.41 to 4.20	Agree	Uninfluential
4.21 to 5	Strongly Agree	Very Uninfluential

The interpretation of these scores differs from typical usage, as it focuses on identifying the factors that contribute to academic weakness rather than overall achievement.

The scale ranged from 5 (Strongly Agree) to 1 (Strongly Disagree). However, for negative statements, reverse scoring was applied to ensure consistency in interpretation.

This reverse scoring was applied to prevent bias and capture a balanced assessment of factors that both positively and negatively influence academic achievement.

In the qualitative phase, twelve questions were designed for interviewing pharmacy students. These questions were designed to elicit precise responses regarding the factors affecting the academic achievement of pharmacy students in Syrian universities.

Data were collected through face-to-face interviews conducted at the participants’ workplaces. The interviews were conducted, transcribed, and coded by the principal investigator (M.Q) and lasted 30-40 minutes on average. A comprehensive SWOT analysis was carried out to evaluate the strengths, weaknesses, opportunities, and threats present among pharmacy student, ^18^ aiming to identify both the advantages and disadvantages and their impact on academic achievement.

### Statistical analysis

Data in the quantitative phase were analyzed using Statistical Package for Social Sciences version 26.0 (SPSS 26) (IBM Corp. Released 2019. IBM SPSS Statistics for Windows, Version 26.0. Armonk, NY: IBM Corp). ^19^ It is confirmed that a valid copyright license for the use of SPSS version 26.0 has been obtained. Microsoft Excel may be used as an open-access alternative. Descriptive statistics were applied to compute means, frequencies and percentages for the responses to the questionnaire items. To measure the reliability of the questionnaire, Cronbach’s alpha coefficient was employed.

The interviews in the qualitative phase were transcribed verbatim, and were analyzed using thematic content analysis.

Thematic analysis of the detailed interview notes was performed using open coding to identify key concepts, which were then organized into emerging themes.

## Results

### Quantitative phase results

**Demographic characteristics of the participating students**

The questionnaire was completed by a total of 1008 pharmacy students, with 773 females and 235 males. The percentage of female pharmacy students constituted (77%) of the total sample size, while the percentage of male students was (23%), which reflects the female-to-male proportion in pharmacy faculties in Syria.

There were 127 (12.6%) second-year students, 271 (26.9%) third-year students, and 243 (24.1%) fourth-year students. The highest proportion of students was in the fifth year, with 367 (36.4%) of the total sample. Data were collected from 23 Syrian Universities, consisting of 6 public universities, and 17 private universities. The number of participating students from public universities was 499 (49.5%), while 509 (50.5%) students, representing private universities of the total sample.

**Descriptive statistics**

Table 2 summarizes the results of the descriptive analysis.

**Table 2.** Results of Questionnaire.

Items	Strongly Agree	Agree	Neutral	Disagree	Strongly Disagree	Mean	Remarks
**Factors related to the student**						**3.17**	
1. My social life is good.	192(19%)	608(60.3%)	137(13.6%)	63(6.3%)	8(0.8%)	3.91	Uninfluential
2. My admission to the pharmacy faculty was in accordance with my parents' wishes. *	98(9.7%)	143(14.2%)	181(18%)	470(46.6%)	116(11.5%)	3.36	Neutral
3. I receive sufficient support from my family in my studies.	423(42%)	449(44.5%)	95(9.4%)	31(3.1%)	10(1%)	4.23	Very Uninfluential
4. I have the ability to manage my time well.	16(1.6%)	100(9.9%)	207(20.5%)	369(36.6%)	316(31.3%)	2.14	Influential
5. In faculty, I am able to ask the questions I want.	118(11.7%)	377(37.4%)	272(27%)	207(20.5%)	34(3.4%)	3.34	Neutral
6. I am able to concentrate well on learning.	104(10.3%)	373(37%)	388(38.5%)	126(12.5%)	17(1.7%)	3.42	Uninfluential
7. Working while attending university is an additional burden and negatively affects learning. *	57(5.7%)	334(33.1%)	265(26.3%)	241(23.9%)	111(11%)	3.01	Neutral
8. I find it difficult to study regularly. *	329(32.6%)	461(45.7%)	115(11.4%)	91(9%)	12(1.2%)	2.00	Influential
**Factors related to the Lecturer**						**3.06**	
9. The lecturers in the pharmacy faculty have good learning skills.	133(13.2%)	440(43.7%)	323(32%)	97(9.6%)	15(1.5%)	3.57	Uninfluential
10. The lecturer explains the scientific material in a good and clear manner.	94(9.3%)	456(45.2%)	349(34.7%)	93(9.2%)	16(1.6%)	3.51	Uninfluential
11. The lecturer provides me with suitable and effective learning opportunities	69(6.8%)	252(25%)	293(29.1%)	359(35.6%)	35(3.5%)	2.96	Neutral
12. The lecturer treats all students fairly.	89(8.8%)	396(39.3%)	282(28%)	180(17.9%)	61(6.1%)	3.27	Neutral
13. There is sufficient motivation from the lecturers.	52(5.1%)	178(17.7%)	278(27.6%)	435(43.2%)	65(6.4%)	2.72	Neutral
14. The lecturer responds to students' questions during lectures.	157(15.6%)	629(62.4%)	187(18.6%)	29(2.9%)	6(0.6%)	3.89	Uninfluential
15. The role of the lecturer is indoctrinated. *	289(28.7%)	346(34.3%)	243(24.1%)	104(10.3%)	26(2.6%)	2.82	Neutral
16. The lecturers attend regularly and are absent only for compelling reasons.	358(35.5%)	522(51.8%)	94(9.3%)	31(3.1%)	3(0.3%)	4.19	Uninfluential
17. There is no interest from the lecturers in the students' academic problems. *	274(27.2%)	356(35.3%)	232(23%)	117(11.6%)	29(2.9%)	2.28	Influential
18. The lecturer uses traditional teaching methods. *	352(34.9%)	381(37.8%)	200(19.8%)	63(6.3%)	12(1.2%)	2.01	Influential
**Factors related to the educational environment**						**2.50**	
19. Cheating is considered a problem in the pharmacy faculty. *	152(15.1%)	210(20.8%)	258(25.6%)	345(34.2%)	43(4.3%)	2.92	Uninfluential
20. There is discrimination among students based on gender. *	82(8.1%)	148(14.7%)	194(19.2%)	449(44.5%)	135(13.4%)	3.40	Uninfluential
21. Lectures are consecutive without breaks. *	187(18.6%)	282(28%)	337(33.4%)	182(18.1%)	20(2%)	2.57	Neutral
22. Teaching in the pharmacy faculty is teacher-centered. *	319(31.6%)	310(30.8%)	224(22.2%)	140(13.9%)	15(1.5%)	2.23	Neutral
23. There is a repetition of topics between courses. *	368(36.5%)	339(33.6%)	157(15.6%)	137(13.6%)	7(0.7%)	2.08	Neutral
24. Some practical courses are taught purely theoretically manner. *	654(64.9%)	266(26.4%)	42(4.2%)	42(4.2%)	4(0.4%)	1.49	Uninfluential
25. Classrooms are not adequately prepared to deliver scientific material. *	358(35.5%)	181(18%)	183(18.2%)	246(24.4%)	40(4%)	2.43	Neutral
26. The practical laboratories are well- equipped.	94(9.3%)	208(20.6%)	183(18.2%)	420(41.7%)	103(10.2%)	2.77	Uninfluential
27. The university provides internet and electronic resources for academic and study purposes.	33(3.3%)	69(6.8%)	111(11%)	388(38.5%)	407(40.4%)	1.94	Influential
28. The educational process is student-centered.	99(9.8%)	274(27.2%)	243(24.1%)	315(31.3%)	77(7.6%)	3.00	Neutral
29. The faculty administration aims for long-term and continuous learning.	75(7.4%)	304(30.2%)	269(26.7%)	315(31.3%)	45(4.5%)	3.05	Neutral
30. The objectives of the educational program are clear.	53(5.3%)	265(26.3%)	270(26.8%)	371(36.8%)	49(4.9%)	2.90	Neutral
31. The curriculum in the pharmacy faculty aims to develop skills in students, not just knowledge.	62(6.2%)	179(17.8%)	180(17.9%)	309(30.7%)	278(27.6%)	2.44	Influential
32. The educational curriculum in the faculty of Pharmacy aims to develop the behavior of pharmacy students.	63(6.3%)	165(16.4%)	214(21.2%)	296(29.4%)	270(26.8%)	2.46	Influential
33. The curriculum in the Pharmacy faculty lacks modernity. *	465(46.1%)	290(28.8%)	147(14.6%)	85(8.4%)	21(2.1%)	1.92	Influential
**Factors related to the Exams**						**2.53**	
34. During exams, I feel extreme stress. *	277(27.5%)	410(40.7%)	198(19.6%)	107(10.6%)	16(1.6%)	2.18	Influential
35. I feel anxious when I have to prepare for exams. *	282(28%)	488(48.4%)	154(15.3%)	75(7.4%)	9(0.9%)	2.05	Influential
36. I only study during the final exam period. *	206(20.4%)	353(35%)	191(18.9%)	230(22.8%)	28(2.8%)	2.52	Influential
37. I strive in my studies to achieve excellent grades, not just success.	322(31.9%)	362(35.9%)	189(18.8%)	118(11.7%)	17(1.7%)	3.85	Uninfluential
38. Exams in the pharmacy faculty only measure the student's memorization. *	393(39%)	337(33.4%)	132(13.1%)	131(13%)	15(1.5%)	2.05	Influential
39. Exam questions are set in a clear and comprehensive manner.	50(5%)	162(16.1%)	264(26.2%)	442(43.8%)	90(8.9%)	2.64	Neutral
40. The final exam in the Pharmacy faculty is the sole criterion for evaluating students. *	245(24.3%)	300(29.8%)	154(15.3%)	226(22.4%)	83(8.2%)	2.61	Neutral
41. Exams in the pharmacy faculty are unfair. *	271(26.9%)	286(28.4%)	268(26.6%)	154(15.3%)	29(2.9%)	2.39	Influential
**Factors related to the Syrian Crisis**						**1.96**	
42. The electricity crisis hinders my learning process. *	390(38.7%)	439(43.6%)	103(10.2%)	68(6.7%)	8(0.8%)	1.87	Influential
43. There are transportation difficulties to and from the university. *	462(45.8%)	350(34.7%)	106(10.5%)	82(8.1%)	8(0.8%)	1.83	Influential
44. The economic conditions negatively impact my learning process. *	372(36.9%)	391(38.8%)	149(14.8%)	88(8.7%)	8(0.8%)	1.98	Influential
45. The war had a negative psychological impact on my academic achievement. *	259(25.7%)	279(27.7%)	295(29.3%)	157(15.6%)	18(1.8%)	2.40	Influential
46. Some male students fail exams to gain a longer deferral period for mandatory service. *	383(38%)	410(40.7%)	182(18.1%)	26(2.6%)	7(0.7%)	1.87	Influential
47. Residential instability affects academic performance. *	374(37.1%)	390(38.7%)	175(17.4%)	55(5.5%)	14(1.4%)	1.95	Influential
48. The crisis causes a decline in motivation towards learning. *	398(39.5%)	428(42.5%)	124(12.3%)	49(4.9%)	9(0.9%)	1.85	Influential

* Scoring was reversed for negative statements.

### Influence student-related factors

The weighted mean for factors related to the students was 3.17.

The statement 3 *“I receive sufficient support from my family in my studies”* had the highest mean at 4.23.

Majority of students 60.3% agreed that their social life is good *“My social life is good”* with 608 students selecting this option. The mean for this statement was 3.91.

However, the statement 2 *“My admission to the pharmacy faculty was in accordance with my parents’ wishes”* had the highest mean among negative statements at 3.36, with 470 respondents selecting the option “disagree”.

In contrast, the statement 8 *“I find it difficult to study regularly”* had the lowest mean of 2.00, with 45.7% agreeing and 32.6% strongly agreeing.

### Influence lecturer-related factors

The weighted mean for the factors related to the lecturer was 3.06.

The statement 16 *“The lecturers attend regularly and are absent only for compelling reasons”* had the highest mean at 4.19, with 51.8% agreeing and 35.5% strongly agreeing. Majority 62.4% agreed that the lecturer responds to students’ questions during lectures, with a mean of 3.89. (Statement 14).

The statement 18 *“The lecturer uses traditional teaching methods”* had the lowest mean at 2.01, with 37.8% agreeing and 34.9% strongly agreeing.

Negative responses were highest for the statement 13 *“There is sufficient motivation from the lecturers”* 43.2% and second highest 35.6% for statement 11 *“The lecturer provides me with suitable and effective learning opportunities”.*

### Influence of educational environment factors

The weighted mean for factors related to educational environment was equal to 2.50. The statement 24 *“Some practical courses are taught purely theoretically manner”* had the lowest mean at 1.49. Similarly, *“The curriculum in the pharmacy faculty lacks modernity”* has a mean of 1.92.

Findings indicated a strong agreement that teaching in the pharmacy faculties is teacher centered.

The statement 28 *“The educational process is student-centered”* received disagreement from 31.3% of the sample responded.

### Influence exams-related factors

The weighted mean of the factors related to the Exams was 3.06.

When asked about exam-related anxiety in the statement 34 *“During exams, I feel extreme stress”*, 40.7% of students agreed, and 27.5% strongly agreed, with a mean of 2.18.

For the statement 35 *“I feel anxious when I have to prepare for exams”,* 48.4% agreed, and 28% strongly agreed, resulting in a mean of 2.05. And the statement 38 *“Exams in the pharmacy faculty only measure the student’s memorization ability”* where the mean for both statements was 2.05, the lowest in this section.

The statement 37 *“I strive in my studies to achieve excellent grades, not just success”* had the highest mean among all the statements in this section 3.85, with 35.9% agreeing and 31.9% strongly agreeing.

### Factors related to the Syrian Crisis

The weighted mean was equal to (1.96). In the statement 42 *“The electricity crisis hinders my learning process”* the sample individuals who agreeing was 43.6%, and the percentage who strongly agreeing was 38.7%, and the mean for this statement was 1.87.

Similarly, in the statement 43 *“There are transportation difficulties to and from the university”* the mean for this statement was 1.83, and the students strongly agreeing with 45.8%, and agreeing with 34.7%. It is worth mentioning that public and not private universities are located in the center of the cities in Syria.

The responses “strongly agree” and “agree” dominated most of the statements in this section, except for the statement 45 *“The war had a negative psychological impact on my academic achievement”* where the highest percentage of responses was “neutral” at 29.3%, with a mean of 2.40.

### Reliability of the questionnaire

Table 3 indicates results of the Cronbach’s alpha coefficient for each of the study variables. Cronbach’s Alpha value for all the statements was 0.892, which is considered as “good”.

**Table 3.** Internal consistency of the study survey instrument.

Measured variable	No of items	Cronbach’s alpha value
Factors related to the student	8	0.526
Factors related to the Lecturer	10	0.810
Factors related to the educational environment	15	0.819
Factors related to the Exams	8	0.585
Factors related to the Syrian crisis	7	0.807
**All variables of the study**	**48**	**0.892**

### Qualitative phase results

The number of participating pharmacy students in the interviews was 30, with 17 female students and 13 male students, where 9 students from public universities, and 21 students from private universities.

### Theme 1: Students perceptions of academic success

Satisfaction with academic achievement: A significant number of students (18 students) expressed their dissatisfaction with their academic achievement. [ *Now, I am at the end of my journey in the faculty before graduation, and when I look back at my academic status, I feel disappointed with what I have accomplished*].

Some students agreed on their dissatisfaction with their academic achievement despite having a good cumulative GPA. [ *Answering this question may be deceiving to the students themselves and others. It is easy to say that I feel satisfied with my academic achievement based on my cumulative GPA in university. However, I feel fearful of the post-graduate stage and lack confidence in my readiness to enter the workforce*].

On the other hand, (8 students) expressed satisfaction with their academic achievement. [ *My self-satisfaction with my academic achievement is very good, considering the circumstances we have gone through and continue to experience during our study period, in addition to the available resources and opportunities at this stage*].

Some other students, specifically four students, expressed that they do not have a clear feeling toward their academic achievement. [ *I cannot say that my academic achievement is bad after all these years in the faculty, but it is also not excellent*].

Motivation: Some students mentioned that their main motivation to achieve good academic performance in the faculty is the ambition to pursue post-graduate studies after graduation. Therefore, maintaining motivation without any slack was crucial, and most of them emphasized the importance of self-motivation in achieving good academic success. [ *When a student has a goal they aspire to reach they will remain in a state of continuous pursuit until they achieve what they desire*].

Two students emphasized the significant benefits derived from the “peer learning” system in maintaining their motivation towards learning. [ *Regular studying with peers is the best way to avoid boredom and the monotony of studying. Through this method, students can maintain their motivation*].

### Theme 2: Facing academic challenges

Some of students pointed out the absence of academic guidance within the faculty, which is considered an important pillar in the success of the educational process.

The majority of responses focused on seeking assistance through various means, such as consulting professors or reaching out to students from previous batches. [ *In every obstacle we faced in the faculty, we would turn to previous students to see how they have dealt with it*].

Another method for seeking help was peers, as some students mentioned benefiting from “peer learning” when confronted with academic challenges. [ *I seek assistance from my peers by regularly meeting and studying together for subjects that we find difficult. We use group discussions to exchange ideas and opinions*].

Time management: In response to the question regarding time management between classes and exam preparation during the semester, the answers varied significantly depending on the university, particularly due to the differences in the exam systems between public and private universities in Syria.

Regarding public university students, there was consensus in the answers about the lack of regularity in studying and preparing for exams throughout the semester. They mentioned that the exam preparation usually starts during the designated exam break at the university. [ *I always prefer to postpone studying for exams until the exam break because I find it sufficient. Preparing for the subjects throughout the semester would be exhausting*].

As for the participating students from private universities (21 students), the majority did not deny their inability to manage their time effectively despite their commitment to studying. The answers revealed differences in methods of preparation and studying for subjects during the semester, but all of them agreed on striving throughout the semester. [ *I try to allocate the majority of my time for studying and exam preparation. I also set aside a small portion of time each day to study the given lessons to avoid accumulation. However, there is still a lack of ability to manage time effectively due to the constant pressure*].

Exam preparation strategies: The disparities in responses among public and private university students persisted, accompanied by varying study strategies influenced by the exam system within each university.

Some students indicated in their answers the “peer learning” system. [ *I prefer studying regularly with some of my friends in faculty. We divide the topics among ourselves, share ideas, and summarize them so that they are ready for the exam period].*

Another group mentioned that they do not give exams significant importance in their study of the subject matter, as much as they focus on the relevance and usefulness of the information in their future careers. [ *Once you study the lectures as soon as you receive them, you can understand and memorize them at the same time. Therefore, studying with the aim of understanding the subject matter and using it practically in the future is considered studying and preparing for the exam*].

Balancing responsibilities: The majority of students emphasized the prioritization of academic responsibilities, indicating their consistent efforts to establish their priorities with a focus on learning. [ *I always strive to prioritize my commitments around learning and allocate a significant amount of time for it. Often, I defer less significant activities for later*].

Furthermore, the students discussed the negative impact of working while studying on academic performance, noting that many students resort to employment due to adverse economic conditions in Syria. [ *My concentration was solely on learning, without pursuing employment or engaging in extracurricular activities. However, I started working due to the poor economic conditions, and I cannot deny that work has had a negative impact on my academic performance].*

### Theme 3: Building a path to excellence: SWOT analysis

Participants were asked about their strengths, weaknesses, opportunities, and threats, aiming to explore the internal and external factors that might affect their academic achievement.

Strengths and weaknesses: Regarding strength factors, many students mentioned their ability to comprehend scientific material well and excel in university exams. [ *One of my strengths is stepping out of the receiver’s circle and relying on research and investigation of each topic, studying references and international books, and comparing the scientific material with what is offered to pharmacy students worldwide*].

Another student who considered time management as a strength point stated, [ *Properly managing my time helps me increase productivity, focus, and achieve set goals, positively impacting my academic performance and success*].

Some students emphasized the significance of reading scientific research papers to improve academic achievement, [ *It can help students to enhance their understanding of the subject matter, increases concentration and attention levels, and strengthens research, analysis, and critical thinking skills*].

However, when it comes to weaknesses, factors such as time management emerged as prominent challenges. [ *I struggle with delaying tasks and often succumb to laziness*].

Another weakness highlighted was difficulty in maintaining focus during lectures. [ *Lack of concentration while attending lectures is a major weakness for me*].

Additionally, some students expressed their concern about exam-related anxiety and its negative impact on their academic performance. [ *Anxiety during exams affects my memory and prevents me from properly recalling information and concepts, resulting in low grades*].

Opportunities and Threats: Regarding opportunities, participants in the interviews highlighted the importance of technology in providing pharmaceutical information quickly and efficiently. [ *I use the internet to search within reliable scientific pages and global academic references to access accurate information that improves my understanding of scientific material*].

The role of lecturers in providing suitable and effective learning opportunities for pharmacy students was emphasized. Lecturers who possess good teaching and learning skills can motivate students and enhance their learning experience. However, some students expressed dissatisfaction with the teaching methods employed within the faculty. [ *There is no doubt that lecturers in the faculty of pharmacy possess knowledge and good teaching skills, but at the same time, they are constrained within one method, and the educational process is teacher-centered*].

Moreover, the economic crisis faced by the country was mentioned by most students as an external factor that significantly affects students’ academic achievements. Additionally, the educational environment and its role in influencing students’ academic performance were discussed. [ *Laboratories in pharmacy faculties were considered a fundamental aspect of the educational process that needs to be well-equipped*].

The curriculum was a topic discussed in many responses, emphasizing its importance in students’ academic achievements. [ *The current curriculum leads students to study great amount of information that are sometimes repetitive, which is challenging in achieving good academic performance*].

Table 4 presents the findings of the participants in the interviews, according to the SWOT analysis.

**Table 4.** SWOT analysis.

**Strengths** • Time management skills. • Motivation for excellence. • Proficiency in scientific subjects. • Active participation with the lecturer. • Reading and conducting research.	**Weaknesses** • Ineffective time management. • Exam-related anxiety. • Lack of focusing during lectures. • Not achieving satisfactory grades despite regular studying.
**Opportunities** • Family support. • Utilization of technological tools in learning. • Lecturers with effective teaching and learning skills.	**Threats** • Challenges related to the Syrian Crisis. • Problems in Pharmacy curriculum design. • Learning is teacher-centered. • Deficiency of the educational environment.

### Theme 4: Future vision of academic success

The participants were asked about their current goals for improving their academic performance and how they plan to leverage academic achievements to fulfill future objectives.

Current goals: The responses were correlated with the answers to the questions regarding strengths and weaknesses in academic performance.

Students who struggle with time management expressed their aspirations to improve their time management skills.

Some students also indicated that their main goal each semester is to improve their cumulative GPA. [ *I know that the cumulative GPA is not the sole measure of academic achievement, but I see it as an important indicator of my academic performance, and I aspire to improve it*].

Among the 30 responses, there were 12 answers that identified training in a pharmacy as a primary goal to be achieved during the year. These students have a moderate to good academic level. Additionally, 8 students mentioned that their main goal for the year is to develop their skills in scientific research, reading a good amount of pharmaceutical research papers to increase their scientific knowledge and improve their academic achievement.

Future goals: Most of the answers were related to the goal of achieving high academic achievement is to continue learning in postgraduate studies or to obtain scholarships after graduation. [ *After completing my years at the faculty of Pharmacy, I aspire to obtain a scholarship, and continue my academic pursuit of learning*] *.*

The importance of academic achievement in obtaining good job opportunities was also discussed. [ *Today, the number of pharmacy faculty graduates is very high, and in order to secure a good job opportunity in the field of pharmacy, you need to have an advantage over other students, and that can be achieved through high academic achievements*].

## Discussion

In medical education, mixed-methods research is ideal for advancing theoretical frameworks, and contributing significantly to enhancement of learning. ^20^

This mixed-methods research study employed quantitative surveys and qualitative interviews to explore the factors affecting the academic achievement of pharmacy students in Syrian public and private universities.

The value of Cronbach’s alpha in the quantitative phase which were considered as good (0.892) provided evidence about the reliability of the applied 5-point Likert scale. ^21^

In the qualitative phase, thematic analysis was selected for this study due to its accessibility and flexibility, as it offers a structured approach to coding and analyzing qualitative data while allowing link to broader theoretical or conceptual aspects. ^22^

In this study, SWOT analysis framework was utilized to assess their own strengths, weaknesses, opportunities, and threats in order to plan their academic journey effectively. ^23^ Furthermore, SWOT strategies help educational leaders in realizing their plans and identifying effective approaches to overcome potential barriers. ^24^

The findings obtained from students both quantitatively and qualitatively, highlighted a common issue of ineffective time management skills identified as a major weakness negatively impacting their academic achievement.

Conversely, students who demonstrated effective time management skills acknowledged the positive impact of this skill on academic performance.

This study concluded that students’ motivation toward learning is a crucial factor in improving academic performance. It was found that lack of motivation from professors was prevalent among students, with self-motivation playing a more significant role.

These results align with the study by ^25^ which highlighted the critical role of grit—defined as perseverance and self-motivation—in the academic success of pharmacy students across 14 Asian and Middle Eastern countries. Similar to the Syrian context, where students largely relied on intrinsic motivation, the study suggests that the ability to persist in the face of academic challenges significantly impacts performance, especially in resource-constrained settings. ^25^

Additionally, family support was identified as another important factor influencing students’ motivation towards learning and improving academic achievement.

Previous studies have indicated the influence of exams on academic performance. ^5^^,^^9^^,^^13^ In this study, exam-related anxiety and exam competence have emerged as factors that contribute to the academic weaknesses observed among pharmacy students and their correlation with academic achievement.

Findings of this research indicate that studying strategy of pharmacy students in Syria is linked to the type of educational system (public or private) in Syrian universities. Factors such as lack of persistence in learning and postponing academic tasks were noted as contributors to decreased academic achievement.

Students expressed concerns about mentors lack of attention to their educational challenges and suggested that strengthening academic counseling within pharmacy faculty could be a viable solution.

While students generally praised professors for their teaching skills, and commitment to attendance, they felt that the provision of suitable and effective learning opportunities by professors was lacking. The students perceived the educational environment in pharmacy faculties as teacher-centered, which they believed hinders the learning process and affects the academic performance negatively.

Other studies have addressed peer learning and its role in enhancing academic performance. ^6^ In this study, some students favored the peer learning system for enhancing student motivation and addressing academic challenges within the faculty.

This study highlighted issues affecting the academic achievement of pharmacy students in Syrian universities. Problems in curriculum design, lack of skill development and unclear program objective were identified.

The pharmacy curricula mainly focus on knowledge acquisition, neglecting skill development and competency building. Repetitive information, theoretical teaching methods and limited practical training were noted.

Students addressed the negative impact of poorly equipped classrooms and laboratories on academic performance.

Students recognized the importance of utilizing technological tools to improve academic achievement with the internet research being a frequent practice. This finding aligns with a previous study, where students acknowledged the significance of employing technological tools to enhance academic performance. ^26^

The findings were consistent with previous findings which reported that academic performance of students is influenced by external factors such as insufficient resources, and socioeconomic factors. ^27^ The Syrian crisis, including economic, social, and psychological challenges, negatively affected academic achievement of pharmacy students with electricity and transportation crises hindering the learning process.

Students expressed motivation towards achieving high academic performance, aiming to continue learning after graduation and pursue applying for scholarships.

Based on the findings and discussions presented in this study, several steps are recommended to enhance the academic achievement of pharmacy students in Syrian universities. First, the content of curricula and educational programs within pharmacy colleges at both governmental and private universities should be reevaluated to improve the learning process and boost students’ academic performance. Additionally, a comprehensive strategy should be developed to address university-related issues and challenges within the educational environment that may impact student learning and academic success. This includes activating the role of academic counseling within pharmacy colleges to ensure proper guidance and support for students facing academic challenges. Professors should also be supported in implementing modern teaching methods to provide suitable and effective learning opportunities. Furthermore, the promotion of peer learning among students is essential to increase motivation and facilitate the exchange of expertise, skills, and knowledge. Training courses for faculty members should be organized to enhance their ability to design and assess exams accurately. It is also crucial to leverage technological resources and the internet to support the learning process, scientific research, and students’ academic performance. Finally, the study recommends conducting in-depth research on the differences in factors influencing academic achievement between governmental and private universities in Syria.

### Research limitations

Differences in the educational systems, evaluation methods, and sources of motivation posed challenges in accurately measuring student motivation and study strategy.

Public and private universities’ grading systems varied, impacting the study’ outcome. The students participating in the questionnaire and the interview were not asked about their cumulative GPA, due to the difference in the system for calculating student grades between public and private universities. Private universities rely on the student’s cumulative GPA to evaluate them, while public universities adopt the final grades system and presented as a percentage.

The difference in sources of motivation between internal motivation, which comes from the student himself, and external motivation, which the student obtains through family, teachers, and peers, and the difference in the reasons motivating the student towards the learning process between each student and another, has caused difficulty in measuring Motivation accurately.

## Conclusion

The results of the mixed-methods study indicated that personal factors related to time management and motivation towards learning have an impact on academic achievement. Exams-related anxiety, as well as the Syrian crisis also play a significant role in weakening academic achievement. Furthermore, factors related to lecturers, learning methods, and the educational environment all have an influence on the academic achievement of pharmacy students in Syrian universities.

### Ethical considerations

Ethical approval for this study was obtained from the Ethical Committee of the Syrian Virtual University (Approval Number: 233/o, Date: 13/2/2023). This study adheres to the ethical principles outlined in the Declaration of Helsinki.

Informed, written consent was obtained from all participants prior to completing the survey forms in the quantitative phase, and verbal consent was recorded before conducting the interviews in the qualitative phase. Participants’ information and data were treated with strict confidentiality, ensuring that personal details were anonymized. The collected data were securely stored on a password-protected computer, and measures were taken to dispose of the data appropriately once it was no longer needed.

The decision to use verbal consent instead of written consent in the interviews was approved by the ethics committee based on the following reasons:

Enhanced participant comfort and flexibility: Verbal consent was considered more comfortable for participants and provided greater flexibility in the study participation process, as they were not required to sign a written document.

Preservation of participant anonymity: Obtaining verbal consent was the preferred option to maintain participant anonymity and minimize potential risks associated with written documentation.

The use of verbal consent was ethically approved by the committee, and all necessary precautions were taken to ensure the confidentiality and protection of participants’ rights.

The quantitative phase of the study was conducted and reported in accordance with the Strengthening the Reporting of Observational Studies in Epidemiology (STROBE) guidelines. ^16^

The qualitative phase was designed and reported according to the Consolidated criteria for Reporting Qualitative research (COREQ). ^17^

## Data Availability

Mendeley Data: “Enhancing Academic Success: A mixed Study on the Influencing Factors among Pharmacy Students in Syrian Universities”. https://doi.org/10.17632/6r46mdszng.4. ^28^ This project contains the following underlying data: COREQ.checklist.pdf Ethical Approval.pdf Responses of Pharmacy Students to Questionnaire.csv Qualitative Interviews.pdf Data are available under the terms of the Creative Commons Attribution 4.0 International license (CC-BY 4.0). Mendeley Data: STROBE checklist cross-sectional study of “Enhancing academic success: A mixed study on the influencing factors among pharmacy students in Syrian Universities”. https://doi.org/10.17632/5zm3vh5kn2.1. ^16^ Mendeley Data: COREQ checklist for “Enhancing Academic Success: A mixed Study on the Influencing Factors among Pharmacy Students in Syrian Universities”. https://doi.org/10.17632/k4f472b3pp.2. ^17^ Data are available under the terms of the Creative Commons Attribution 4.0 International license (CC-BY 4.0).

## Software availability

The following software programs were utilized in the research process:

SPSS 26 (IBM Corp. Released 2019. IBM SPSS Statistics for Windows, Version 26.0. Armonk, NY: IBM Corp). ^19^

Microsoft Excel may be used as an open-access alternative for basic data management.

For downloading Microsoft Excel, you can visit the official Microsoft website at https://www.microsoft.com/en-us/microsoft-365/excel.

The references were managed using Zotero software https://www.zotero.org/download/. ^29^
