# Supplementary material for: Enhancing Academic Success: A mixed Study on the Influencing Factors among Pharmacy Students in Syrian Universities
Source: F1000Res. 2024 Oct 11;13:868. Originally published 2024 Aug 1. [Version 2] doi: 10.12688/f1000research.151218.2 (PMC11525093; doi:10.12688/f1000research.151218.2)
Supplement: Peer review history

# Reviewer report: Reviewer response for version 2

**DOI:** 10.5256/f1000research.172320.r330862

**Published:** 2024-10-30

**Authors:** Naeem Mubarak

**Affiliations:** 1 Department of Pharmacy Practice, University of Health Sciences, Lahore Medical & Dental College, Lahore University of Biological & Applied Sciences, Pakistan

The revised manuscript is approved for indexing

Is the work clearly and accurately presented and does it cite the current literature?

Yes

If applicable, is the statistical analysis and its interpretation appropriate?

Yes

Are all the source data underlying the results available to ensure full reproducibility?

Yes

Is the study design appropriate and is the work technically sound?

Yes

Are the conclusions drawn adequately supported by the results?

Yes

Are sufficient details of methods and analysis provided to allow replication by others?

Yes

Reviewer Expertise:

NA

I confirm that I have read this submission and believe that I have an appropriate level of expertise to confirm that it is of an acceptable scientific standard.

# Reviewer report: Reviewer response for version 2

**DOI:** 10.5256/f1000research.172320.r330863

**Published:** 2024-10-16

**Authors:** Leah Li Echiverri

**Affiliations:** 1 Wenzhou-Kean University, Wenzhou, China

After careful review, I am pleased to inform you that the revisions have addressed the concerns raised, and the paper is now approved.

Is the work clearly and accurately presented and does it cite the current literature?

Yes

If applicable, is the statistical analysis and its interpretation appropriate?

Partly

Are all the source data underlying the results available to ensure full reproducibility?

Yes

Is the study design appropriate and is the work technically sound?

Yes

Are the conclusions drawn adequately supported by the results?

Partly

Are sufficient details of methods and analysis provided to allow replication by others?

Yes

Reviewer Expertise:

Curriculum and Instruction, Teaching and Learning, Artificial Intelligence Integration,  Faculty Training and Development, English as a Second Language (ESL),  English as Foreign language (EFL), Student Learning, Student Satisfaction/Motivation, Academic Performance

I confirm that I have read this submission and believe that I have an appropriate level of expertise to confirm that it is of an acceptable scientific standard.

# Reviewer report: Reviewer response for version 1

**DOI:** 10.5256/f1000research.165848.r315175

**Published:** 2024-09-09

**Authors:** Naeem Mubarak

**Affiliations:** 1 Department of Pharmacy Practice, University of Health Sciences, Lahore Medical & Dental College, Lahore University of Biological & Applied Sciences, Pakistan

**Enhancing Academic Success: A mixed Study on the Influencing Factors among Pharmacy Students in Syrian Universities**

**Verdict: Minor Revision**

 Thank you for providing me the opportunity to review this interesting read. The study explores the factors associated with academic success among pharmacy students in Syrian universities. The study addresses a crucial topic with depth and detail, however, following are some recommendations to further improve the impact of the study.

**Introduction**

1. Describe what constitutes academic achievement in context to the objectives of your study
2. The introduction should focus on the various aspects and factors that influence academic achievement in Syrian universities. It is essential to emphasize why pharmacy students were specifically chosen for this study. To strengthen the evidence regarding the factors affecting the academic progress of pharmacy students across different continents, the authors may consider citing the following study (This is optional and should only be taken as a suggestion for the improvement of the manuscript) Elnaem MH.et.al.,2024 (Ref 1)

**Methodology**

1. Was there any inclusion/ exclusion criteria with respect to the recruitment of students, public and private universities, geographical limitations of data collection etc?    
2. Why first year pharmacy students were not made part of the study as their academic progress and achievement were highly influenced by the factors explored under the study which could have resulted in robust data collection. Please justify
3. What was the targeted sample size and response rate achieved during the data collection?
4. "Additionally, three academic members reviewed the questionnaire to assess its clarity and relevance of the statements in alignment with the study’s aims" The authors should mention the criteria chosen for the selection of experts for content and face validity.
5. How the questionnaire was modified after the idea generation phase? How many statements were added/deleted and modified?
6. The result interpretation for the five point likert scale in Table 1 creates confusion especially while analyzing the reverse coded items. How can the authors reach to this interpretation of the extent of influence without establishing a correlation of the factors with the academic achievement

**Discussion**

 The discussion appears as the repetition of the findings. More concrete synthesis of the findings are needed to evaluate the findings. Comparison with the existing literature and studies in other low and middle income countries would establish the profound impact of the study. You may cite the following updated references. (This is optional and should only be taken as a suggestion for the improvement of the manuscript)

 Elnaem MH.et.al.,2023 (Ref 2)

Is the work clearly and accurately presented and does it cite the current literature?

Yes

If applicable, is the statistical analysis and its interpretation appropriate?

Yes

Are all the source data underlying the results available to ensure full reproducibility?

Yes

Is the study design appropriate and is the work technically sound?

Yes

Are the conclusions drawn adequately supported by the results?

Yes

Are sufficient details of methods and analysis provided to allow replication by others?

Yes

Reviewer Expertise:

pharmacy practice and policy

I confirm that I have read this submission and believe that I have an appropriate level of expertise to confirm that it is of an acceptable scientific standard, however I have significant reservations, as outlined above.

## References

[1] : Assessment of Academic Resilience and Its Associated Factors Among Pharmacy Students in Twelve Countries. *Am J Pharm Educ* .2024;88(5) : 10.1016/j.ajpe.2024.100693 100693 10.1016/j.ajpe.2024.100693 38574997

[2] : Evaluation of grit and its associated factors among undergraduate pharmacy students from 14 Asian and Middle Eastern countries amid the COVID-19 pandemic. *Saudi Pharm J* .2023;31(3) : 10.1016/j.jsps.2023.01.008 410-416 10.1016/j.jsps.2023.01.008 36744104 PMC9890879

# Response

**Published:** 2024-09-22

**Authors:** Mohab Qattan

**Affiliations:** Medical Education, Syrian Virtual University, Damascus, Damascus Governorate, Syria

**Dear reviewer Naeem Mubarak**

 Thank you for your thorough review of our manuscript. We are grateful for the time and effort you have dedicated to providing constructive feedback. We would like to assure you that each comment will be addressed point by point. Your insights are invaluable, and we are committed to incorporating your suggestions to enhance the overall quality of the research.

**Introduction**

- **Describe what constitutes academic achievement in context to the objectives of your study**
  Thank you for your valuable feedback. We acknowledge the importance of clearly defining academic achievement in the context of our study's objectives. In our revised manuscript, we have provided a detailed explanation of how academic achievement is operationally defined, specifically as it relates to the performance of pharmacy students. This definition focuses not only on exam results but also on factors such as student engagement, the ability to apply knowledge in practical settings, and overall progression through their academic programs.

- **The introduction should focus on the various aspects and factors that influence academic achievement in Syrian universities. It is essential to emphasize why pharmacy students were specifically chosen for this study. To strengthen the evidence regarding the factors affecting the academic progress of pharmacy students across different continents, the authors may consider citing the following study (This is optional and should only be taken as a suggestion for the improvement of the manuscript) Elnaem MH.et.al.,2024 (Ref 1)**
  We appreciate your suggestion to provide further emphasis on the factors influencing academic achievement in Syrian universities and the rationale for focusing on pharmacy students. In the revised introduction, we have expanded the discussion to highlight the unique challenges faced by pharmacy students in Syria. We also added a justification for selecting pharmacy students. Additionally, we considered your recommendation and cited the study by Elnaem MH.et.al.,2024 (Ref 1) to offer a broader perspective on the academic progress of pharmacy students across different regions.

**Methodology**

1. **Was there any inclusion/ exclusion criteria with respect to the recruitment of students, public and private universities, geographical limitations of data collection etc?    **
  Thank you for highlighting this point. In our revised methodology section, we have clarified the inclusion and exclusion criteria for the recruitment of participants. Specifically, pharmacy students from both public and private universities were included to ensure a representative sample across different educational systems. Geographical limitations were minimal, as students from universities across various regions in Syria were invited to participate, ensuring diverse input.
2. **Why first year pharmacy students were not made part of the study as their academic progress and achievement were highly influenced by the factors explored under the study which could have resulted in robust data collection. Please justify**
  We appreciate your observation regarding the exclusion of first-year pharmacy students. The decision to exclude these students was deliberate, as the first academic year in Syria, particularly at universities such as Damascus University, is commonly referred to as the "preparatory year." During this year, students are not fully committed to a specific faculty, as they can choose to enter Medicine, Dentistry, or Pharmacy at the end of the year based on their academic grades.
  Since first-year students have not yet made a firm decision regarding their specialization, their academic progress does not fully reflect the dynamics of pharmacy education.
  Additionally, the decision was based on the assumption that first-year students may not have had enough exposure to the full range of factors affecting academic achievement, such as exam-related anxiety or curriculum structure, which become more prominent in the later stages of study.
  In summary, while recognizing the potential insights that could be gained from including first-year students, the exclusion was made to maintain the study’s focus and ensure a more comprehensive understanding of academic achievement factors among students who are fully immersed in pharmacy education.
3. **What was the targeted sample size and response rate achieved during the data collection?**
  Thank you for your valuable observation. We have added the information regarding the targeted sample size and the response rate in the revised version of the manuscript.
4. **"Additionally, three academic members reviewed the questionnaire to assess its clarity and relevance of the statements in alignment with the study’s aims" The authors should mention the criteria chosen for the selection of experts for content and face validity.**
  Thank you for your insightful feedback. We have now clarified the criteria for the selection of the experts who reviewed the questionnaire in the revised version of the manuscript. 
5. **How the questionnaire was modified after the idea generation phase? How many statements were added/deleted and modified?**
  Thank you for pointing out the need for further clarification. After the idea generation phase, the questionnaire underwent a series of modifications based on feedback from the expert reviewers. Initially, the questionnaire contained 52 items. Following the review process, 6 items were removed due to redundancy and lack of clarity, while additional 2 items were added to better address the educational environment and exam factors, bringing the final number of items to 48. Several other items were modified to enhance clarity and ensure that the statements were fully aligned with the study’s objectives. These changes were made to improve the overall validity and reliability of the questionnaire.
  In the revised manuscript, we have elaborated on the modifications made to the questionnaire after the idea generation phase.
6. **The result interpretation for the five point likert scale in Table 1 creates confusion especially while analyzing the reverse coded items. How can the authors reach to this interpretation of the extent of influence without establishing a correlation of the factors with the academic achievement**
  We appreciate your concerns regarding the interpretation of the five-point Likert scale, especially for reverse-coded items. In response, we have provided a more detailed explanation in the revised manuscript. We have clarified how reverse scaling was employed to prevent response bias, and we have addressed the importance of interpreting these items in relation to academic weakness rather than achievement. While we acknowledge that establishing direct correlations between factors and academic achievement would provide additional depth, this was beyond the scope of the current study, which focused on exploring perceived influences.
**Discussion**
**The discussion appears as the repetition of the findings. More concrete synthesis of the findings are needed to evaluate the findings. Comparison with the existing literature and studies in other low and middle income countries would establish the profound impact of the study. You may cite the following updated references. (This is optional and should only be taken as a suggestion for the improvement of the manuscript)**
** Elnaem MH.et.al.,2023 (Ref 2)**
  Thank you for your valuable feedback. We appreciate your suggestion regarding the discussion section. In response, we have revised the discussion to provide a more concrete synthesis of the findings and included a comparison with relevant literature from studies conducted in other low- and middle-income countries. We have also incorporated the suggested reference by Elnaem MH et al., 2023, to strengthen the discussion and highlight the global relevance of our study’s findings.

# Reviewer report: Reviewer response for version 1

**DOI:** 10.5256/f1000research.165848.r310497

**Published:** 2024-08-23

**Authors:** Leah Li Echiverri

**Affiliations:** 1 Wenzhou-Kean University, Wenzhou, China

Observation: Based on Table 1, the result interpretation for the weighted mean in assigning values is somewhat counterintuitive because typically, a higher score (e.g., 4.21 to 5) on a Likert scale would indicate a stronger positive agreement or influence. However, in this table, the highest weighted mean range (4.21 to 5) is interpreted as "Very Uninfluential," which contrasts with the usual interpretation of "Strongly Agree" as having a strong positive influence. 

 Comment 1:

 The scale seems to be reversed or designed for a specific context where disagreement with a statement corresponds to a positive influence, and agreement corresponds to a negative influence. If this scale is applied to positive statements, it may confuse readers or participants because the typical expectation is that "Strongly Agree" correlates with a strong positive impact.

 Furthermore, if you apply this table's interpretation to a reversed scale for negative statements, the results could become misleading. A lower score (indicating agreement with a negative statement), such as item 8 for example, would be interpreted as "Influential" using this table, but normally, agreement with a negative statement should be seen negatively.

 The reversed scale in the table creates a significant shift in how results are interpreted. If using this table, it's essential to clearly explain the reversal and how it impacts the interpretation, especially when dealing with reversed scoring for negative statements. Otherwise, it could lead to confusion and misinterpretation of the data.

 Comment 2: Based on the quantitative and qualitative findings, provide recommendations for enhancing academic success with a focus on areas that need improvement (particularly those items with low means.)

Is the work clearly and accurately presented and does it cite the current literature?

Yes

If applicable, is the statistical analysis and its interpretation appropriate?

Partly

Are all the source data underlying the results available to ensure full reproducibility?

Yes

Is the study design appropriate and is the work technically sound?

Yes

Are the conclusions drawn adequately supported by the results?

Partly

Are sufficient details of methods and analysis provided to allow replication by others?

Yes

Reviewer Expertise:

Curriculum and Instruction, Teaching and Learning, Artificial Intelligence Integration,  Faculty Training and Development, English as a Second Language (ESL),  English as Foreign language (EFL), Student Learning, Student Satisfaction/Motivation, Academic Performance

I confirm that I have read this submission and believe that I have an appropriate level of expertise to confirm that it is of an acceptable scientific standard, however I have significant reservations, as outlined above.

# Response

**Published:** 2024-09-22

**Authors:** Mohab Qattan

**Affiliations:** Medical Education, Syrian Virtual University, Damascus, Damascus Governorate, Syria

**Dear reviewer Leah Li Echiverri**

 Thank you very much for your detailed review and valuable comments. We apologize for the delayed response, as we were awaiting the second review report requested by the journal's editorial team.

**Observation: Based on Table 1, the result interpretation for the weighted mean in assigning values is somewhat counterintuitive because typically, a higher score (e.g., 4.21 to 5) on a Likert scale would indicate a stronger positive agreement or influence. However, in this table, the highest weighted mean range (4.21 to 5) is interpreted as "Very Uninfluential," which contrasts with the usual interpretation of "Strongly Agree" as having a strong positive influence.**

**Comment 1:**

**The scale seems to be reversed or designed for a specific context where disagreement with a statement corresponds to a positive influence, and agreement corresponds to a negative influence. If this scale is applied to positive statements, it may confuse readers or participants because the typical expectation is that "Strongly Agree" correlates with a strong positive impact.**

**Furthermore, if you apply this table's interpretation to a reversed scale for negative statements, the results could become misleading. A lower score (indicating agreement with a negative statement), such as item 8 for example, would be interpreted as "Influential" using this table, but normally, agreement with a negative statement should be seen negatively.**

**The reversed scale in the table creates a significant shift in how results are interpreted. If using this table, it's essential to clearly explain the reversal and how it impacts the interpretation, especially when dealing with reversed scoring for negative statements. Otherwise, it could lead to confusion and misinterpretation of the data.**

 Regarding your observations, I would like to clarify the interpretation of the results and the data presented in Table 1. The inclusion of both positive and negative statements in the scale was intentional to prevent bias in participant responses. The interpretation was based on the question: "What are the factors that contribute to weaknesses in academic achievement?"

 For example, a positive statement such as "I receive sufficient support from my family in my studies" elicited responses predominantly in agreement, which indicated that this factor was perceived as very uninfluential on academic weakness. Conversely, for a statement like "I have the ability to manage my time well," responses tended towards disagreement, suggesting that this factor was influential in contributing to academic weakness.

 This design enabled us to assess both positive and negative influences on academic weakness, rather than on overall academic achievement. We acknowledge that the reversal of scoring might be unconventional, and we have provided a more detailed explanation of the reverse scaling in the manuscript to ensure a clear understanding of how this impacts data interpretation. Our aim was to offer a balanced assessment of factors impacting academic achievement by focusing on their contribution to academic weaknesses.

 Thank you again for your valuable feedback, which helped us improve the clarity and depth of our study.

**Comment 2: Based on the quantitative and qualitative findings, provide recommendations for enhancing academic success with a focus on areas that need improvement (particularly those items with low means.)**

 Thank you for your insightful comment and guidance. Your feedback was truly appreciated, and recommendations were formulated based on your guidance.
